# Defining the Angolan Highlands Water Tower, a 40 plus-year precipitation budget of the headwater catchments of the Okavango Delta

**DOI:** 10.1007/s10661-023-11448-7

**Published:** 2023-06-19

**Authors:** Mauro Lourenco, Stephan Woodborne

**Affiliations:** 1 School of Geography, Archaeology and Environmental Studies, University of the Witwatersrand, Johannesburg, South Africa; 2 National Geographic Okavango Wilderness Project, Wild Bird Trust, Johannesburg, South Africa; 3 iThemba LABS, Private Bag 11, Johannesburg, South Africa

**Keywords:** Okavango Delta, Peatlands, Remote sensing, Google Earth Engine, Flood

## Abstract

Angola is a source of many major rivers in southern Africa and is referred to as the “water tower” of the region. The lack of a defined area delineating the Angolan Highlands water tower (AHWT) limits the conservation of this important freshwater source. This study hydrologically defines the boundary of the AHWT as areas > 1274 m above mean sea level within the Central Bié Plateau of Angola. Using the Climate Hazards Group InfraRed Precipitation with Station (CHIRPS) data, this study provides a 41-year precipitation budget of the AHWT and surrounding basins. Between 1981 and 2021, the average annual precipitation over the AHWT was 1112 mm and the gross annual average precipitation volume was approximately 423 km^3^ over an area of 380,382 km^2^. The AHWT is the southern source of the Congo Basin, the western source of the Zambezi Basin, and the sole water source of the endorheic Okavango Basin and Okavango Delta, a UNESCO World Heritage Site. On average, approximately 133 km^3^ (92.36%) of the gross annual precipitation volume for the headwater Cuito and Cubango catchments of the Okavango River is lost before reaching the Okavango Delta. Estimates of the annual flooding of the Okavango Delta during a 35-year period (1985–2019) were correlated to precipitation in the headwater catchments. Correlation coefficients are stronger for the entire rainfall season (0.76) and early rainfall season (0.62) for the combined Cuito–Cubango catchment in comparison to late rainfall season (0.50), which suggests that the antecedent conditions (first and second flood pulse) during the early rainfall season allows for greater Okavango Delta flood inundation. The correlation coefficients between the Cubango (0.72) and Cuito (0.78) Rivers and annual flood inundation are not significantly different (*P* > 0.05); however, these rivers have fundamental hydrological differences that influence the functioning of the Okavango Delta. The Cubango River, described as a flushing system, has much steeper gradient, more compact and shallow soils and flows faster with significant rapids, whereas the peatland rich, absorbent, seepage-driven baseflow of the Cuito River sustains the Okavango Delta during the dry season. The dynamics of seasonal precipitation, hydrology and climate change in the AHWT have important repercussions on water budgets, food security and biodiversity throughout southern Africa, requiring continued collaboration between countries to ensure that future development is sustainable.

**Supplementary Information:**

The online version contains supplementary material available at 10.1007/s10661-023-11448-7.

## Introduction

Angola is the ‘water tower’ for much of southern and south-central Africa (Huntley, 2019). The region is the source of nine large hydrographic basins, seven of which are transboundary (Huntley, 2019). These river systems originate from the interior Bié Plateau of the Angola Highlands and arise on either side of undulating watersheds that define large river catchments such as the Cuanza, Cassai (Congo), Lungui-Bungu (Zambezi), Cunene, Cuito and Cubango (Okavango: Huntley, 2019; Lourenco et al., 2022a). The Angolan Highlands water tower (AHWT) is the primary source of the Okavango Delta, designated as the 1000^th^ site to be officially inscribed on the UNESCO World Heritage Site List on 22 June 2014 (UNESCO, 2014; Yurco et al., 2017). Water flows into the Okavango River from two tributaries: the Cuito and Cubango Rivers (Gumbricht et al., 2004; McCarthy et al., 2000). As is the case in all the major catchments, the annual flood of the Okavango Delta is dependent on upstream precipitation (Inman & Lyons, 2020; Wolski et al., 2017), that considerably influences its ecological and hydrological functioning (Van Wilgen et al., 2022). Despite this importance, the Angolan Highlands and its water tower have received much less attention than the Okavango Delta.

Highland regions, throughout the world play a major role in determining global and regional climates, are the source of most rivers, support biodiversity, are refuges and bridges for species, and are crucial for the sustainability of multiple human societies (Capitani et al., 2019; Perrigo et al., 2020). In semi-arid and arid regions, highlands play an important role in providing freshwater resources (Chen et al., 2016). Southern Africa is considered an arid region and is projected to become generally drier under low-mitigation climate change futures (Archer et al., 2018). Climate change is likely to lead to greater variability and an overall decrease of available freshwater, while human water use is likely to increase owing to population and economic growth (Bernauer & Böhmelt, 2020; Capitani et al., 2019). Scientific and policy debates over human impacts on global freshwater resources have intensified, particularly in the context of growing concerns about the implications of climate change on vulnerable freshwater systems (Bernauer & Böhmelt, 2020). Moreover, drought occurrence has increased in the Angolan Highlands (Lourenco et al., 2022a) and is intensifying in frequency and severity in southern Africa more broadly, presenting challenges to long-term water and food security in the region (Gore et al., 2020; Nhamo et al., 2019).

The extensive minefields resulting from the Angolan Civil War from 1975 to 2002 limited access to the isolated Angolan Highlands region, and despite its hydrological and ecological significance, it is little studied (Carvalho et al., 2017; Lourenco et al., 2022b). Using the Climate Hazards Group InfraRed Precipitation with Station (CHIRPS) data, the objectives of this study are to (1) define the extent of the Angolan Highlands and its water tower, (2) provide a precipitation budget of the AHWT and surrounding catchments and (3) provide the relationship between the timing and amount of precipitation in the Angolan Highlands source catchments and flooding of the Okavango Delta.

## Methods

### Study site

**Fig. 1:**
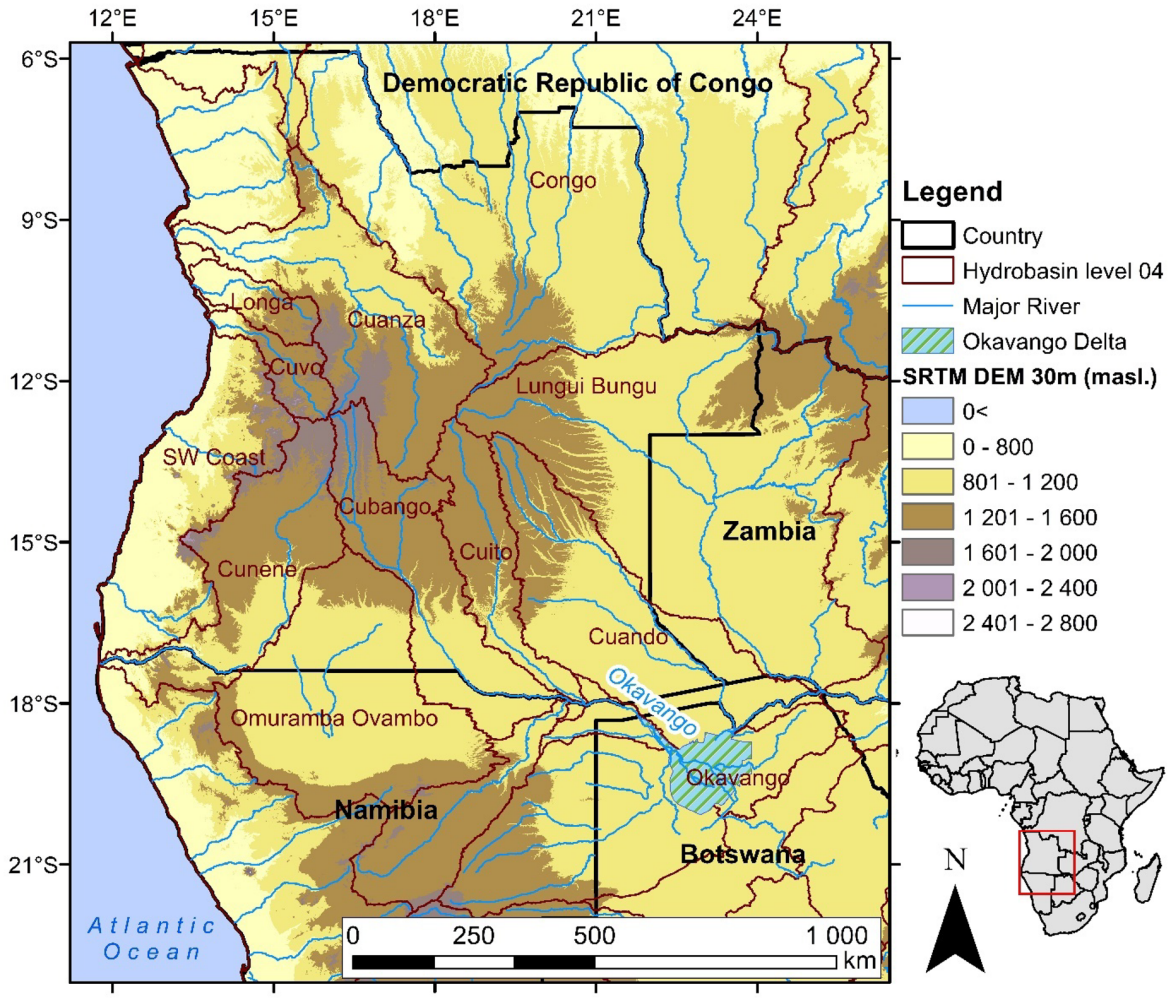
Topography of Angola, including the WWF HydroSHEDS Basins Level 04 (data from Lehner and Grill, 2013) and major river basins originating from the Bié Plateau of Angola including the Okavango River and the extent of the Okavango Delta

Angola has a distinct topography (Fig. 1), ranging from the western coastal lowlands below 200 m above sea level (masl) that occupy a band between 10 and 150 km in width and cover 5% of the country, to areas above 1500 masl that cover 7% of the country (Huntley, 2019). Adjacent to the coastal lowlands, the western mountainous escarpment rises to 1000 masl and covers 23% of Angola (Huntley, 2019). The plateau is 1000–1500 masl and covers over half (65%) of Angola (Huntley, 2019). The central highlands of the Bié Plateau are the source water for major drainage basins including the Cuanza, Congo, Zambezi and Okavango (Lourenco et al., 2022a). The Okavango River catchment is shared by Angola, Namibia and Botswana (McCarthy et al., 2003), and it is estimated that over 95% of the water that flows into the Okavango Delta originates from precipitation in the Angolan Highlands (Folwell et al., 2006). Precipitation in the Angolan Highlands is seasonal (September–April) with peak rainfall occurring in January (Lourenco et al., 2022a). This rainwater collects in source lakes that feed the main river channels (Conradie et al., 2016). The highland river systems are surrounded by miombo woodlands, tropical and subtropical grasslands, tree and shrub savannas (Goyder et al., 2018). The river valleys are characterised by seepages, oxbow lakes, wet grasslands and peatland deposits (Goyder et al., 2018; Lourenco et al., 2022b).

### Defining the Angolan Highlands water tower

The term ‘highland’ is used synonymously with ‘mountain’ (Ives, 2001) and refers to a relatively mountainous or elevated part of a country or region (Perrigo et al., 2020). There is no universally accepted definition of ‘mountain’, nor a definition or characterisation of a ‘highland’ (Ives, 2001). Delineation according to elevation is inconsistent, for example the Icelandic Highlands are situated above 400–500 masl (Eddudóttir et al., 2020), whereas the Lesotho Highlands are situated above 3400 masl (Knight & Grab, 2022). Over annual scales, highlands tend to be cooler, wetter versions of the climates of nearby lowlands (Perrigo et al., 2020). ‘Water towers’ refer to the water supply and storage that mountain ranges provide to support and sustain environmental and human water demands downstream (Immerzeel et al., 2020). Water towers are recognised by their buffering capacity, as water stored in snow, glaciers (cryospheric water towers) and lakes provide a relatively constant supply of water to downstream areas (Immerzeel et al., 2020). African water towers have been identified according to their elevation, precipitation and contribution of water resources to regions and populations far beyond their delineated boundaries (UNEP, 2010). Much like most of Africa’s water towers, the AHWT is not cryospheric, the year-round buffering is supplied by source lakes and minerotrophic peatland deposits (Lourenco et al., 2022b). The peatlands are a control valve between ground water flow supplied by seasonal precipitation and the river flow (Lourenco et al., 2022b). Considering the importance of the Bié Plateau for water resources, the delineation of the elevation that defines the AHWT is derived from precipitation data.

**Fig. 2:**
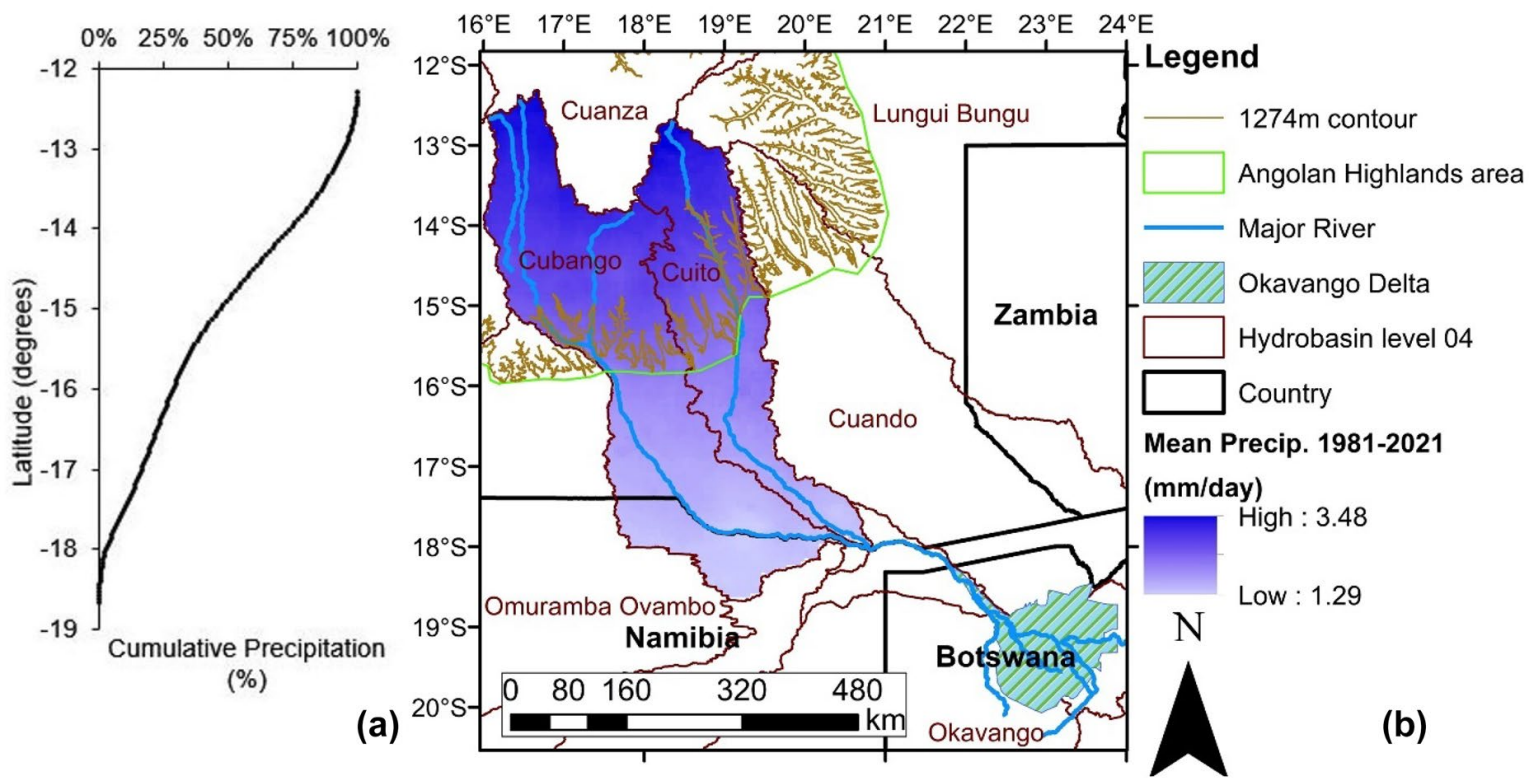
(**a**) The cumulative sum of the individual CHIRPS precipitation pixels (increasing from south to north) according to latitude over the Cuito and Cubango catchments. (**b**) The spatial distribution of mean daily CHIRPS precipitation (1981–2021) over the Cuito and Cubango catchments including the 1274 masl contour line, the AHWT area and the Okavango Delta

Average annual precipitation is approximately 1300 mm/year in the Angolan Highlands (Lourenco et al., 2022a) in comparison to the highly seasonal rainfall (November–March: 300–500 mm/year) of the Okavango Delta (McCarthy et al., 2000, 2003). The Okavango Delta lies within the semi-arid Kalahari Desert of Botswana, where potential evaporation is estimated to be over 2000 mm/year (Gumbricht et al., 2004). The annual Okavango Delta flood event occurs out of phase with the rainfall season as the inundated area expands from a low of 3500–5000 km^2^ in January–February to a high of 6000–11,000 km^2^ in July–September during the late dry season (Gumbricht et al., 2004; Inman & Lyons, 2020; McCarthy et al., 2003; Wolski et al., 2017). This is due to the large distance (600 km) between the headwater source, the slow propagation of the flood within the Okavango Delta itself (McCarthy et al., 2003) and extensive peatlands in the Angolan Highlands that buffer baseflow in the headwaters (Lourenco et al., 2022b). A rainfall gradient exists within the Cuito and Cubango catchments (Fig. 2), with approximately 50% of the cumulative sum of daily precipitation across the Cuito and Cubango catchments occurring north of 14° 47′ S latitude. The average elevation at that latitude across the Cuito and Cubango catchments is 1274 masl. Therefore, we delineate the AHWT to be the areas within the central Angolan Bié Plateau that are above 1274 masl. Due to the backward erosion and incision caused by the river systems, a neat boundary line was drawn around the 1274 masl contour line to delineate the AHWT area.

### Precipitation data

The Angolan network of meteorological weather stations collapsed during the civil war, and as a result, studies of Angola’s climate have been hindered over the past four decades due to a lack of reliable climatic data (Huntley et al., 2019). A minimum of 30 years of climatic data is needed to derive higher order statistics and trends, as shorter records are often influenced by modes of climate variability that obscure a long-term trend (World Meteorological Organization, 2017). The precipitation data for the study has been collected from a remote sensing (RS) product, as historical data longer than 30 years, specific to this study area, are unavailable. Historical precipitation data for the region were collated using the Google Earth Engine (GEE) platform. Precipitation data were obtained from the CHIRPS 30 + year quasi-global rainfall dataset (Funk et al., 2015). CHIRPS synthesises 0.05° resolution satellite imagery with in situ station data to create gridded rainfall time series (Funk et al., 2015). On GEE, daily precipitation data was collated for the period spanning 41 years from 1981–01-01 to 2021–12-31. This data was used to produce a map of the spatial distribution of precipitation over the region using ArcMap 10.2; thereafter, this map was used to calculate the annual precipitation budget for the Angolan Highlands and its catchments. In addition, a time series of daily precipitation for the Cuito, Cubango and for the combined Cuito and Cubango catchments (named Cuito–Cubango) were extracted from GEE.

### Precipitation budget

The spatial distribution of mean CHIRPS daily precipitation over the period 1981–01-01 to 2021–12-31 were calculated on GEE and individual pixel values were exported to ArcMap 10.4 (ESRI, 2023) at a 5-km resolution. This mean daily precipitation was clipped to the extent of each of the Hydro basin level 4 catchments. A second clip was performed to clip the spatial precipitation to the individual catchments within the AHWT. Using this mean precipitation data, annual precipitation for each pixel was calculated by multiplying the mean daily rainfall (mm/day) by 365.25 to provide a pixel value in mm/ year. The individual pixel values were converted to m/year by dividing by 1e3, multiplied by the pixel size (m^2^), and then summed together to provide a total rainfall in m^3^ for each catchment, for convenience the total rainfall was converted to km^3^ by dividing by 1e9.

### Relationship between precipitation and Okavango flood inundation

Within the Angolan Highlands, the rainfall season starts in September each year and continues to April, with a dry season from May to August (Lourenco et al., 2022a). For the Cuito, Cubango and combined Cuito–Cubango catchments, the daily rainfall was summed to provide total rainfall in each rainfall season. These totals were compared to five individual studies that provide RS derived estimates of flood inundation (from 1985 to 2019) for the Okavango Delta. The individual flood estimates indicate the annual maximum (July–September out of phase) flood inundation extent of the Okavango (Inman & Lyons, 2020). Different combinations of consecutive rainfall months (early rainfall season: September–December, late rainfall season: January–April and total rainfall season: September–August) from each rainfall year were used to correlate precipitation in the Cuito, Cubango and combined Cuito–Cubango catchments with flood inundation in the Okavango Delta. All statistical analyses were performed using Microsoft XL stat (Lumivero, 2023), and data visualisations were performed in Power BI (Microsoft, 2023).

Annual Okavango flood inundation extent estimates were extracted from five separate studies for the months of July–September (1985–2019) using web plot digitizer (https://apps.automeris.io/wpd/). RS derived inundation maps from each study were produced using different products at different resolutions: National Oceanic and Atmospheric Administration Advanced Very-High-Resolution Radiometer (NOAA AVHRR) 1000 m: McCarthy et al. (2003) and Gumbricht et al. (2004), Moderate Resolution Imaging Spectroradiometer (MODIS) MCD43A4 500 m: Wolski et al. (2017), MODIS MOD09Q 250 m: Thito et al. (2016) and Landsat 30 m: Inman and Lyons (2020). Each of these estimates was used to calculate average inundation extent per year, in some years the average is derived from a single estimate.

### Land cover of the Cuito and Cubango catchments

The European Space Agency (ESA), Climate Change Initiative (CCI), land cover classification (Copernicus Climate Change Service, Climate data store, 2019) and 300-m resolution maps for 2020 were extracted and clipped to the boundaries of the Cuito and Cubango catchments to provide an estimate of the land cover within each catchment.

## Results

### Precipitation budget of the Angolan Highlands

The AHWT, as defined above, occupies an approximate area of 380,382 km^2^ and intersects 11 (eight are transboundary) river catchments according to the WWF HydroSHEDS Basins level 4 product (Lehner & Grill, 2013: Fig. 3). Between 1981 and 2021, gross annual average precipitation volume within the AHWT was calculated to be 423 km^3^. Across the study area, precipitation decreases southwards, the highest mean daily precipitation (5.49 mm/day) from 1981 to 2021 occurred in the Democratic Republic of Congo (DRC). The Congo River basin is the largest (894,498 km^2^); however, only 24,995 km^2^ (2.79%) lies within the AHWT area. High precipitation over the Congo Basin means that the AHWT delineation only applies to the other catchments where lower reaches have reduced precipitation relative to the AHWT. More than half of the area within the Cuito (59.65%) and Cubango (53.23%) catchments lies within the AHWT area, with 65.57% and 64.14% of the precipitation estimated to occur within the AHWT area respectively. The highest (92.72% and 73,28%) proportion of precipitation within the AHWT area occurred within Cuvo and Cunene catchments, respectively.

**Fig. 3:**
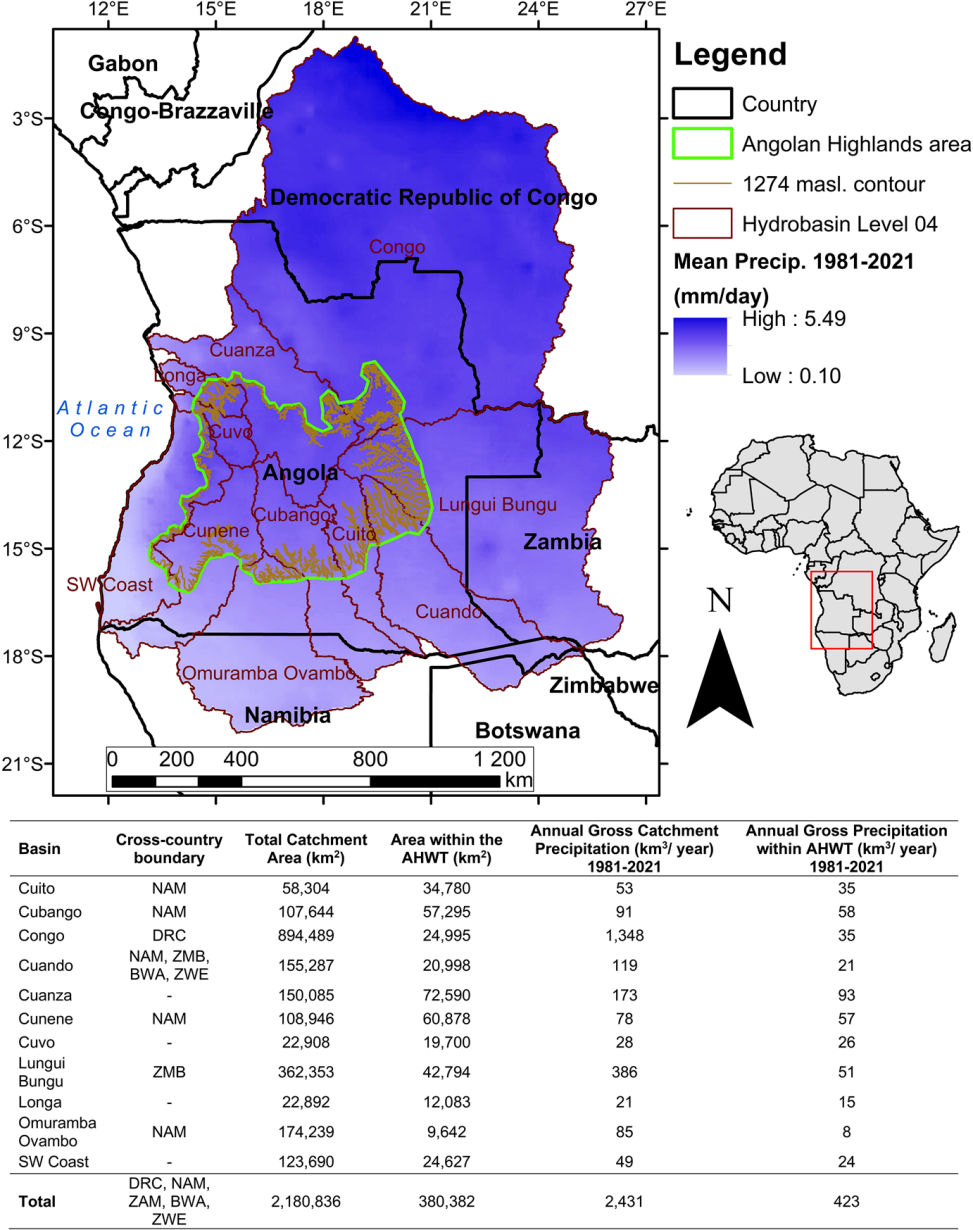
Mean daily precipitation in the AHWT and surrounding Hydrobasin level 04 catchments. Estimated gross annual average precipitation volume (km^3^/year) per catchment and within the AHWT. There are multiple spellings of each river catchment name, the versions presented are the first which were encountered by the authors

### Seasonality in precipitation in the headwater catchments of the Okavango Delta

**Fig. 4:**
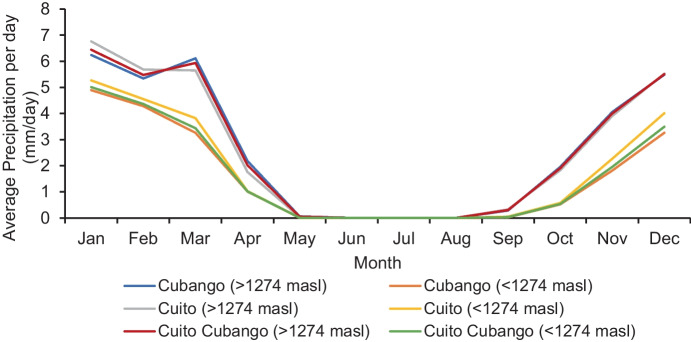
Average daily precipitation per month from 1981 to 2021 in the Cuito, Cubango and combined Cuito–Cubango catchments within the AHWT (> 1274 masl) and below the AHWT (< 1274 masl)

The CHIRPS data illustrates the strong seasonality in precipitation in the headwater catchments of the Okavango Delta (Fig. 4). The mean daily precipitation from 1981 to 2021 is calculated for the Cuito, Cubango and combined Cuito–Cubango catchments for those areas above and below the AHWT boundary line. According to the CHIRPS data, precipitation starts during September each year in the Cuito and Cubango catchments within the AHWT, reaching peak average (Cuito: 6.76 mm and Cubango: 6.24 mm) daily precipitation in January. The average daily precipitation is similar between the Cuito and Cubango catchments within the AHWT, 2.62 mm/day and 2.64 mm/day respectively, but slightly higher in the Cuito compared to the Cubango catchment below the AHWT, 1.80 mm/day and 1.59 mm/day respectively. For the combined Cuito–Cubango catchment, average annual rainfall (1981–2021) for the areas within the AHWT and below the AHWT is 963 mm and 605 mm respectively. The rainfall season lasts through to April and an almost entirely dry season lasts from May to August. The seasonality of precipitation in these catchments is applied to subsequent results. A total of eight rainfall months lasting from September to April is split into two separate rainfall seasons, an early rainfall season from September to December and a late rainfall season from January to April.

### Precipitation time series and Okavango Delta flood inundation

**Fig. 5:**
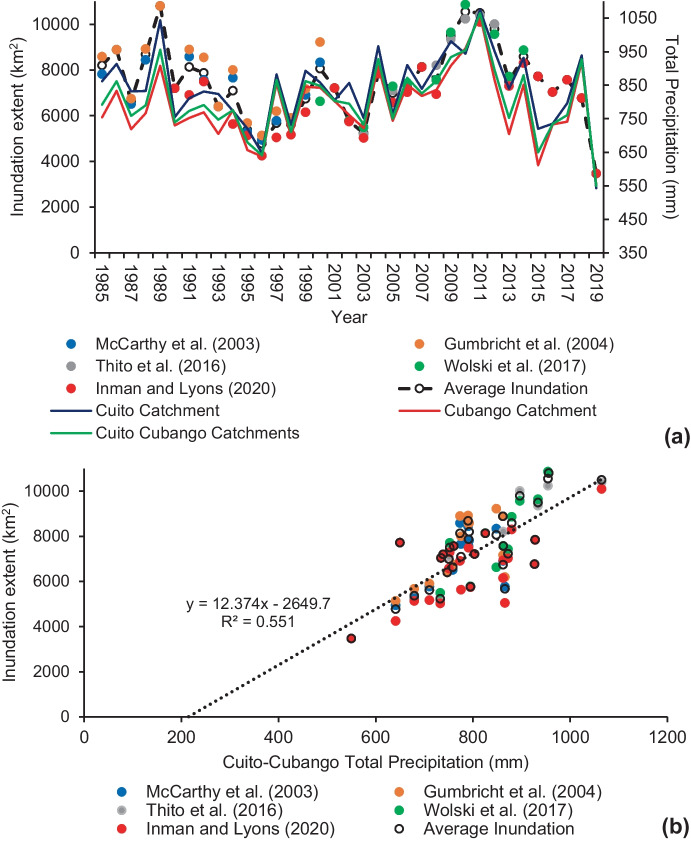
(**a**) Annual out of phase (July–September) flood inundation extent (km^2^) from five separate studies (coloured circles), including the average flood inundation extent (km^2^) per year (white circle with dashed line) and the total precipitation (coloured solid lines) in each rainfall season from the Cuito, Cubango and combined Cuito–Cubango catchments. (**b**) Scatter plot and linear trend line of the annual flood inundation extent from five separate studies and total precipitation in each rainfall season for the combined Cuito–Cubango catchment

Flood inundation estimates of the Okavango Delta spanning the period from 1985 to 2019 were derived from five separate studies (Fig. 5). Total precipitation for each rainfall season (September–August) was calculated for the Cuito, Cubango and combined Cuito–Cubango catchments. For example, the total precipitation for 1985 indicates the rainfall occurring from September 1984 to August 1985. Highest average flood inundation estimates from 1985 to 2019 occurred during 1989 (10,800 km^2^), 2010 (10,551 km^2^) and 2011 (10,497 km^2^) and lowest occurred during 2019 (3470 km^2^), 1996 (4775 km^2^) and 2003 (5232 km^2^). By comparison, highest total precipitation for the combined Cuito–Cubango catchment occurred during 2011 (1065 mm), 1989 (956 mm) and 2010 (954 mm) and lowest occurred during 2019 (550 mm), 1996 (641 mm) and 2015 (650 mm). On average, precipitation in the Cuito catchment was 842 mm per year between 1985 and 2019, by comparison the Cubango catchment was 788 mm per year. See supplementary material Fig. 1 for the total precipitation per year from 1981 to 2021 in the Cuito, Cubango and combined Cuito–Cubango catchments within the AHWT (> 1274 masl) and below the AHWT (< 1274 masl).

### Relationship between precipitation and Okavango flood inundation

Different combinations of consecutive rainfall months (early rainfall season: September–December, late rainfall season: January–April and entire rainfall season: September–August) from each rainfall year were used to correlate precipitation in the Cuito, Cubango and combined Cuito–Cubango catchments with RS derived flood inundation estimates of the Okavango Delta (Table 1). Correlation coefficients are stronger for the full rainfall season and early rainfall season in comparison to late rainfall season for most of the inundation estimates. In addition, correlation coefficients are stronger for the Cuito catchment (0.78) compared to the Cubango (0.72) catchment (although correlation coefficients do not differ significantly) for the average inundation estimate over the entire rainfall season.

**Table 1:**
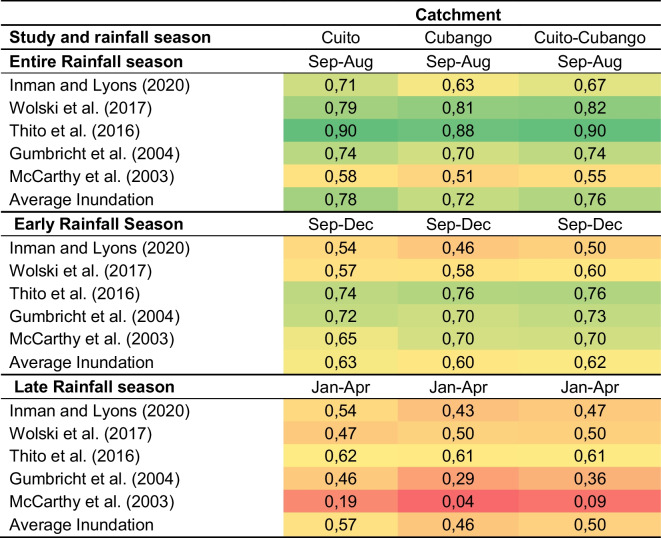
Correlation table between Okavango flood inundation estimates and precipitation within the Cuito, Cubango and Cuito–Cubango catchments from 1985 to 2019. A colour palette is used to emphasise high and low correlation coefficient values

All values presented are different from 0 with a significance level alpha = 0.05 (*p* < 0.005)

### Land cover of the Cuito and Cubango rivers

**Fig. 6:**
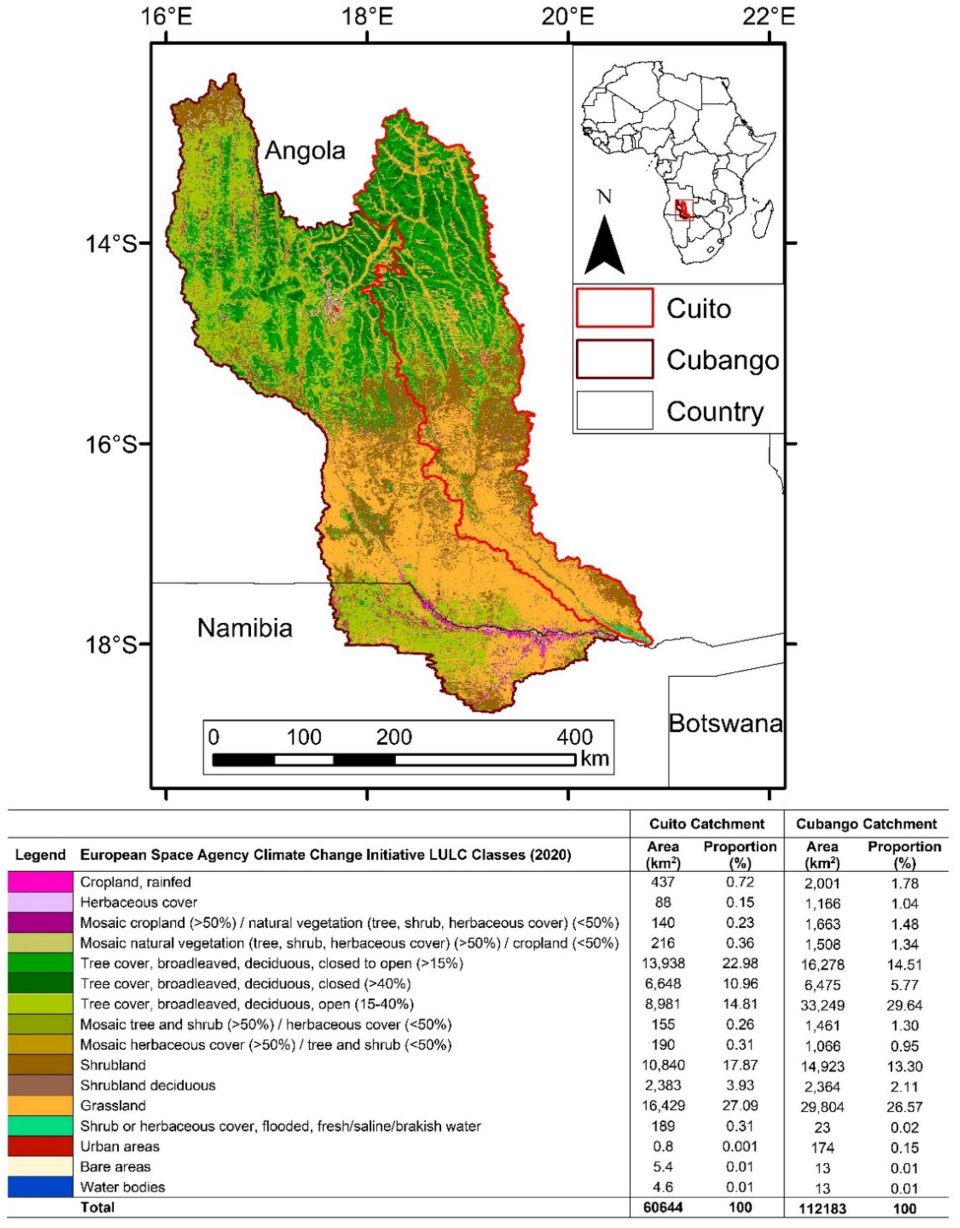
The 2020 300-m resolution ESA CCI land cover area for the Cuito and Cubango catchments

Land cover estimates reveal the Cuito catchment contains 437 km^2^, 88 km^2^, 140 km^2^ and 0.8 km^2^ of area classified as *Croplands, Herbaceous cover, Mosaic croplands and urban areas* respectively. By comparison, the Cubango catchment contains 2100 km^2^, 1166 km^2^, 1663 km^2^ and 174 km^2^ of area classified as *Croplands, Herbaceous cover, Mosaic croplands and Urban areas* respectively (Fig. 6). Although having a larger proportion of total surface area, the Cubango catchment contains a higher proportion (4.46% vs. 1.10%) of land covered by land cover types that influence river discharge. These land cover classes are typically along the border with Namibia where the Cubango River is commonly referred to as the Kavango River in Namibia (OKACOM, 2023).

## Discussion

### The Angolan Highlands water tower

This study is the first to delineate the boundary of the AHWT, which is hydrologically defined to areas > 1274 masl. The precipitation budget from 1981 to 2021 revealed that the gross annual average precipitation volume of 423 km^3^ fell over the AHWT, supporting major river catchments such as the Okavango, Zambezi and Congo which flow into neighbouring countries such as Botswana, Namibia, Zambia, Zimbabwe, Mozambique and the DRC. Angolan Highlands rivers fall into two separate categories. The first comprises coastal rivers that drain the central and western highlands and flow rapidly westwards, penetrating the escarpment and eventually reach the Atlantic Ocean (Huntley, 2019). These coastal rivers are relatively short and energetic, for example the Longa and Cuvo rivers are highly erosive and carry high sediment loads (Skelton, 2019). The Cuanza and Cunene coastal basins in the west formed due to erosion of the escarpment and western interior plateau, whereas the Congo escarpment has been captured by the Congo River from the north (Skelton, 2019). The second category of river systems is that of the interior Bié Plateau, which serves as the ‘water tower’ for much of southern and central Africa (Huntley, 2019).

The main drainage systems of the AHWT include the southern source tributaries of the Congo River, the western source tributaries of the Zambezi River which also flows in to the Chobe River, coastal rivers that flow into the Atlantic, the Cuando River which flows southeast and provides water to the Linyanti Swamps and Suvuti Channel on the border between Botswana and Namibia (Mendelsohn, 2022) and the endorheic Etosha Pan (Cuvelai Basin) in Namibia and Okavango Basin drainages to the south (Huntley, 2019; Skelton, 2019). Angola is an emerging African economy with a rapidly growing human population and increasing demand on freshwater resources (Skelton, 2019). Many Angolan rivers are unregulated; however, there are three major hydroelectric dams on the Cuanza system with a further four being planned on the Cuanza system alone (Skelton, 2019). The end of the Angolan Civil War has brought stability and development to the Angolan Highlands region, increasing the likelihood of water abstractions, pollution, introduction of invasive species and possible reductions in water resources due to changing precipitation and evaporation patterns (Folwell et al., 2006; Mendelsohn & Weber, 2015). The conservation and protection of Angolan river systems is of great significance, particularly for transboundary systems of the Okavango, Congo and Zambezi rivers which support local communities and ecosystems of global importance (Huntley, 2019; Mendelsohn, 2022; Mendelsohn & Weber, 2015; Skelton, 2019).

Angola is particularly vulnerable to climate change (Brooks et al., 2005). Due to the paucity of meteorological stations, climate projection studies rarely include Angola and commonly provide evaluations of climate models over multiple countries (Carvalho et al., 2017). Both stronger and more consistent droughts are expected through the next century (Brooks et al., 2005; Cain, 2015), with generally drier (precipitation decreases of up to 4%) and warmer (increases up to 4.9 °C) conditions expected for the Angolan Highlands region by 2100 (Carvalho et al., 2017). The drought history of the Angolan highlands indicates negative trends in precipitation and increased drought frequency since 1981 (Lourenco et al., 2022a). The 1995–1997 and 2018–2020 drought events coincide with the two lowest average Okavango Delta flood inundation estimates during 1996 (4775 km^2^) and 2019 (3470 km^2^). The year 2019 was documented as the driest year (precipitation total of 550 mm within the combined Cuito–Cubango catchment) on record since 1981, having serious implications on food security and health in the southern provinces of Angola (Lourenco et al., 2022a), this extended downstream to the Okavango Delta.

Rainfall over southern Africa is strongly influenced by climate variability in the Angolan Highlands (Crétat et al., 2019). Synoptic drivers of precipitation in the region, such as the Angola Low, have only recently received strong attention (Crétat et al., 2019; Howard & Washington, 2018). In addition, the drought pattern over southern Africa is not yet fully understood and the role of the Angola Low for moisture transport from the Southern Atlantic remains understudied (Gore et al., 2020). What is recognised is that the Angolan Highlands have hydrological and ecological importance (Gumbricht et al., 2004; McCarthy et al., 2000; Van Wilgen et al., 2022). Future research avenues including hydrological and precipitation monitoring will have strong implications for water budgets (Lourenco et al., 2022a). Precipitation patterns, climate variability and anthropogenically induced climate change in the Angolan Highlands are likely to affect freshwater resources, food security and biodiversity across southern Africa.

### Implications for the Okavango Delta

The Okavango River basin is a transboundary basin with a network of river systems that transverse through Angola, Namibia and Botswana (Inman & Lyons, 2020; Wolski et al., 2017). Between 1981 and 2021, 65.57% and 64.14% of the precipitation fell over the AHWT portion of the Cuito and Cubango catchments respectively. On average, approximately 11 km^3^ of water flows into the Okavango Delta each year (Mosepele et al., 2022), providing water to roughly 1 million people and the permanent and seasonal wetlands of the Okavango Delta which support important biodiversity (Mendelsohn et al., 2010). It is estimated that most (55%) of the water comes from the Cubango River and 45% from the Cuito River (Mendelsohn & el Obeid, 2004). The gross annual average precipitation volume reveals that 91 km^3^ (63.19%) and 53 km^3^ (36.81%) of precipitation falls over the Cubango and Cuito catchments each year respectively. Over the AHWT, the proportion of the Cuito is slightly higher, with 58 km^3^ (62.37%) and 35 km^3^ (37.63%) of precipitation in the Cubango and Cuito catchment respectively. The gross annual average precipitation volume of 144 km^3^ per year over the two headwater catchments provides approximately 11 km^3^ to the Okavango Delta (UNESCO, 2014), meaning that approximately 133 km^3^ (92.36%) of the precipitation is lost to groundwater recharge, evaporation, transpiration or water abstraction.

The Cubango River, with a flow length of 1260 km, has its source in the eastern part of Huambo Province at 1850 masl, whereas the Cuito River (flow length of 920 km) rises between Cangoa and Sachiambe at 1430 masl in the western part of Moxico Province (National Geographic Okavango Wilderness Project: NGOWP, 2018). The significant differences in geomorphology and soil properties between the Cuito and Cubango Rivers are fundamental to the ecological and hydrological functioning of the Okavango Delta (NGOWP, 2018). The Cubango River has a much steeper gradient with more compact and shallow soils than the Cuito River and flows faster with more significant rapids (Baumberg et al., 2014; NGOWP, 2018; Mendelsohn, 2022). The Cubango is described as a flushing system, early rain contributes to the fast-flowing river and reaches the Okavango Delta as the first flood pulse between January and March. The second flood pulse occurs between April and May, attributed predominantly to the Cuito River which is characterised by deep Kalahari sands and slow flow rates (NGOWP, 2018; Mendelsohn, 2022). The upper Cuito catchment consists of meandering rivers in a broad low-gradient patchwork mosaic of wetlands, marshes, floodplains, wet grasslands and peatlands that are flanked by moist miombo forests (NGOWP, 2018; Lourenco et al., 2022b). The contribution of runoff in the Okavango is due to the absorbent, seepage-driven baseflow of the Cuito River (Pröpper et al., 2015). The peatlands of the Cuito are essential to the baseflow contribution into the Okavango Delta, slowing and retaining water during intense rainfall periods in the highlands, releasing the stored water into the Okavango system during the dry season (NGOWP, 2018; Lourenco et al., 2022b).

Precipitation totals for early rainfall, late rainfall and entire rainfall season in the Cuito, Cubango and combined Cuito–Cubango catchments were correlated with five separate RS derived flood inundation estimates for the Okavango Delta. Accuracy assessments of each RS derived inundation estimates reveals that overall accuracy between each study ranges from 89% (McCarthy et al., 2003) to 99.4% (Thito et al., 2016). Correlation coefficients are stronger for the full rainfall season and early rainfall season in comparison to late rainfall season for most of the inundation estimates. In addition, correlation coefficients are stronger for the Cuito catchment compared to the Cubango catchment. This suggests that high rainfall in the early rainfall season (especially within the Cuito catchment) is likely to result in increased Okavango Delta flooding in comparison to high rainfall in the late wet season. In addition, although annual Okavango Delta flood inundation estimates are varied, they are strongly correlated to rainfall during the early rainfall season within the Cuito and Cubango catchments that provide the necessary antecedent conditions (first and second flood pulse) for flooding in comparison to rainfall during the late rainfall season.

### Okavango transboundary river management and conservation

The permanent Okavango River Basin Commission (OKACOM) was established in 1994 to increase cooperation between Angola, Namibia and Botswana with the recognition that future development in the Cubango–Okavango (includes Cuito) River Basin (CORB) is inevitable but must be coordinated to balance the conflicting demands of humans and the ecosystem (Folwell et al., 2006). The CORB supports rural communities that are often located near rivers or along roads. In comparison to the populations of the capital cities and main economic hubs in each country, these remote populations are generally poorer, less healthy and less educated (OKACOM, 2023). This is particularly true in Angola, where historical war slowed social and economic development. The basin is described as one of Africa’s least affected by socio-economic development and remains relatively under-developed (Bybee, 2014; OKACOM, 2023). Potential developments that directly impact water resources, water quality and hydrological flows such as hydroelectric dams and large-scale irrigation schemes have been identified within the basin (OKACOM, 2023); it is essential that such developments are sustainable (Bybee, 2014).

The CORB water audit presents the domestic water use for each country in 1000 s of m^3^ (OKACOM FAO, 2014: Table 2). It is challenging to compare CHIRPS precipitation data with domestic water use as no updated water audits for the CORB are available. Between 2008 and 2011, an average of 134.3 million m^3^ was estimated to be used annually between each country, with 38.67%, 10.43% and 50.90% used by Angola, Botswana and Namibia respectively. Irrigation (58.50%), livestock sector (25.25%) and use in settlements (15.15%) make up 96.90% of the average annual water usage in CORB. The estimated total annual water usage from rivers was 90.1 million m^3^, with Angola (53.09%) and Namibia (42.48%) disproportionally higher than Botswana (4.43%). Table 2 refers to use from water abstractions; it excludes environmental water use and water requirements, water use by wildlife is considered part of environmental use (OKACOM FAO, 2014).

**Table 2:** Average annual (2008–2011) estimated water use per country in the CORB (in 000 s m^3^). Table data is adapted from (OKACOM FAO, 2014)

Water use	Angola	Botswana	Namibia	CORB total
Irrigation	34,825.4	620	43,100	78,545.4
Livestock sector	13,163.8	4900	14,500	32,563.8
Use in settlements	3935.2	6850	8220	19,005.2
Mining		1350		1350.0
Tourism	0.2	280	2530	2810.2
Aquaculture	0.1			0.1
Est. total usage	51,924.7	14,000	68,350	134,274.7
Est. total river water usage	47,825.4	3994	38,270	90,089.4

The river network and its water are an important resource for each country. Described as the best-known feature of the basin, Botswana’s Okavango Delta remains one of the most important areas for biodiversity in the world and makes up most of the country’s tourism industry which generates an estimated 13% of the GDP (Yurco et al., 2017). In Namibia, the river is the main water supply for Rundu and for the commercial irrigation schemes along the Kavango river; augmenting scarce water resources in the country (Folwell et al., 2006; Bybee, 2014). The water use in Namibia is likely to be underestimated as a total of 223 individual water abstractions were identified (74% for agricultural use) on the Namibian side of the Kavango River during 2021, this number increased from 89 in the previous survey during 2017 (NGOWP, 2021). The land cover along the Namibian side of the Kavango River within the 10-km buffer zone includes large portions of areas covered by *Croplands, Herbaceous cover, Mosaic croplands and Urban areas*. The lower correlation coefficients for precipitation in the Cubango catchment in comparison to the Cuito are likely due to the different hydrological characteristics of each river catchment and in part due to the major river abstractions on the Cubango River, as large quantities of rainwater never reach the Okavango Delta.

Population growth, development and the corresponding increase in domestic water supply, and increased irrigation are the main drivers for growing water demand in CORB (Folwell et al., 2006). At the start of the twenty-first century, Angola’s population was 16.39 million, with Namibia and Botswana having a population of 1.82 million and 1.73 million respectively (UN, 2022). By 2022, the population of Angola more than doubled to 35.59 million, whereas Namibia’s population increased to 2.59 million and Botswana to 2.63 million. Population projections for 2050 and 2100 indicate that Angola’s population is likely to double again, potentially reaching 72.33 and 132.90 million by 2050 and 2100 respectively. In contrast, Namibia and Botswana are projected to reach 5.02 and 4.18 million people by 2100 based on the United Nations medium–fertility scenario (UN, 2022). It is critical to ensure that population increase, and subsequent developments are sustainable and do not disrupt the environmental integrity and hydrological functioning of the AHWT. This requires a deep understanding of the AHWT functioning for water resources to surrounding basins. Delineation of the AHWT boundary and an estimation of its water budget is an essential step in facilitating conservation, protection and management strategies of this important water tower within southern Africa.

## Conclusion

The AHWT is a water source region for major rivers of southern Africa. This study is the first to delineate the boundary of the AHWT that is hydrologically defined as areas within the Bié Plateau that are > 1274 masl. The gross annual average precipitation volume of the AHWT is 423 km^3^/year, providing freshwater resources to seven different countries and ecosystems of global importance. On average, approximately 92.36% of the annual precipitation budget for the Cuito and Cubango catchments is lost before reaching the Okavango Delta. A strong positive correlation exists between precipitation in the Cuito and Cubango catchments and flood inundation of the Okavango Delta. Correlation coefficients are stronger for the entire rainfall season and early rainfall season in comparison to late rainfall season, which suggests that the antecedent conditions (first and second flood pulse) provided by high rainfall in the early rainfall season allow for greater Okavango flood inundation. The driest year on record (2019: 550 mm) between 1985 and 2019 coincided with the lowest annual Okavango Delta flood inundation total (3470 km^2^). Steeper gradients, more compact soils, faster flowing waters, rapids and water abstractions along the Cubango River are likely responsible for lower correlation coefficients with Okavango Delta flooding in comparison to the Cuito River. The absorbent, seepage-driven baseflow of the Cuito River provides baseflow to the Okavango Delta during the dry season, facilitated by precipitation during the early rainfall season in the Angolan Highlands. The rivers of the AHWT are largely unregulated and the paucity of literature, because of historical conflict, concerning the hydrological significance of these rivers provides important future research avenues that will have strong implications on water budgets, food security and biodiversity throughout southern Africa. Shared transboundary rivers originating in the AHWT will require continued collaborative efforts to ensure future development is sustainable.

## Supplementary Information

Below is the link to the electronic supplementary material.

Supplementary file1 (DOCX 22 KB)

## Data Availability

The datasets generated during and/or analysed during the current study are available from the corresponding author on reasonable request.
