# Supplementary material for: Defining the Angolan Highlands Water Tower, a 40 plus-year precipitation budget of the headwater catchments of the Okavango Delta
Source: Environ Monit Assess. 2023 Jun 19;195(7):859. doi: 10.1007/s10661-023-11448-7 (PMC10279584; doi:10.1007/s10661-023-11448-7)
Supplement: Supplementary file 1 — Supplementary file1 (DOCX 22 KB) [file 10661_2023_11448_MOESM1_ESM.docx]

**Defining the Angolan Highlands Water Tower, a 40 plus-year precipitation budget of the headwater catchments of the Okavango Delta.**

Mauro Lourenco ^1,2*^ and Stephan Woodborne ^1,3^

^1^ School of Geography, Archaeology and Environmental Studies, University of the Witwatersrand, Johannesburg, South Africa.

^2^ National Geographic Okavango Wilderness Project, Wild Bird Trust, Johannesburg, South Africa.

^3^ iThemba LABS, Private Bag 11, WITS, Johannesburg, South Africa.
*Corresponding author: [mauro@wildbirdtrust.com](mailto:mauro@wildbirdtrust.com)

**Supplementary Material:**

**Fig. 1.** Total precipitation per year from 1981-2021 in the Cuito, Cubango and combined Cuito-Cubango catchments within the AHWT (>1274 masl) and below the AHWT (<1274 masl).
